# Marked Effects of Tachykinin in Myositis Both in the Experimental Side and Contralaterally: Studies on NK-1 Receptor Expressions in an Animal Model

**DOI:** 10.1155/2013/907821

**Published:** 2013-01-29

**Authors:** Yafeng Song, Per S. Stål, Jiguo Yu, Sture Forsgren

**Affiliations:** ^1^Section for Anatomy, Department of Integrative Medical Biology, Umeå University, 901 87 Umeå, Sweden; ^2^Sports Medicine Unit, Department of Surgical and Perioperative Sciences, Umeå University, 901 87 Umeå, Sweden

## Abstract

Muscle injury and inflammation (myositis) in a rabbit model of an unilateral muscle overuse were examined. It is unknown if the tachykinin system has a functional role in this situation. In this study, therefore, the neurokinin-1 receptor (NK-1R) expression patterns were evaluated. White blood cells, nerve fascicles, fine nerve fibers, and blood vessel walls in myositis areas showed NK-1R immunoreaction. NK-1R mRNA reactions were observable for white blood cells and blood vessel walls of these areas. NK-1R immunoreaction and NK-1R mRNA reactions were also seen for muscle fibers showing degenerative and regenerative features. There were almost no NK-1R immunoreactions in normal muscle tissue. Interestingly, marked NK-1R expressions were seen for myositis areas of both the experimental side and the contralateral nonexperimental side. EIA analyses showed that the concentration of substance P in the muscle tissue was clearly increased bilaterally at the experimental end stage, as compared to the situation for normal muscle tissue. These observations show that the tachykinin system is very much involved in the processes that occur in muscle injury/myositis. The effects can be related to proinflammatory effects and/or tissue repair. The fact that there are also marked NK-1R expressions contralaterally indicate that the tachykinin system has crossover effects.

## 1. Introduction

The tachykinins conform to a group of neuropeptides with marked functional roles. The neuropeptide which is most well known in the group is substance P (SP). SP has pronounced pro-inflammatory effects, including the promotion of extravasation and accumulation of leukocytes at sites of injury [1]. SP is also involved in the so-called neurogenic inflammation [2], wound healing [3], and angiogenesis [4]. SP is on the whole known to have autocrine/paracrine effects [5]. SP has a high affinity for the neurokinin-1 receptor (NK-1R), having its major functions via this receptor [6]. The NK-1R has 407 amino acids and belongs to the G-protein-coupled group of receptors [7]. The NK-1R can play an important role in the modulation of the accumulation of white blood cells that occurs in inflammatory processes [8].

There is frequently an upregulation of tachykinins in situations with inflammation and tissue injury. That includes the situation in, for example, ulcerative colitis [9] and experimentally induced acute pancreatitis and associated lung injury [10]. In the latter situation, the NK-1R is considered to play a key role in the damaging process [11]. It is actually a fact that the upregulation of tachykinins that occurs in inflammation in general is associated with increases in NK-1R expression [12, 13].

We have in recent experimental studies been using a rabbit model of a marked overuse affecting the triceps surae muscle and found that the overuse led to muscle inflammation (myositis) and muscle tissue derangements [14]. This model can be considered to be a suitable animal myositis model [14–16]. There is a great lack of information concerning the involvement of tachykinins in situations with myositis and muscle injury. Tachykinins might possibly be involved in the processes that occur. Interestingly, in our studies using the overuse muscle model, it was observed that myositis and muscle tissue derangements not only occurred for the triceps surae muscle in the experimental side but also for this muscle in the nonexercised, contralateral side [14]. This observation of an affection not only for the experimental side but also for the non-experimental contralateral side is completely new information concerning experimentally induced muscle injury/myositis. How the situation is for tachykinins in this respect is therefore also unknown. The information that so far exists concerning tachykinins and muscle inflammation is related to the patterns of SP immunoreactions observed at the spinal cord level and for the neurons innervating the muscles [17–19].

There is no information at all on the expression of the NK-1R in the situation with myositis/marked muscle changes. Our recent observations described above for the overuse rabbit model thus reinforced us to investigate the NK-1R patterns during myositis development. Immunohistochemistry and in situ hybridization were used. Comparisons between tachykinin (SP) and NK-1R expression patterns were also made. The above described experimental model was used, as it has been observed to be suitable in establishing myositis. The triceps surae muscle on both the experimental and non-experimental, contralateral sides were evaluated.

The main aim of the study was to examine for the importance of the tachykinin system in the myositis process. The hypothesis was that NK-1R would be highly expressed in the affected areas with myositis. A further aim was to evaluate if there were upregulations of the NK-1R expressions not only in the experimental side but also in the contralateral non-experimental side.

## 2. Material and Methods

### 2.1. Animals

Female New Zealand white rabbits with a weight of approximately 4 kg (age ranging from 6 to 9 months) were used for the experiment. In total, samples from 24 animals were evaluated. Six of the animals belonged to a reference group (controls). Eighteen animals corresponded to animals that were subjected to an exercise protocol leading to a marked overuse of the triceps surae muscle. They underwent the experimental exercise procedure on their right leg every second day, for a total period of 1, 3, and 6 weeks, respectively, (see further below).

### 2.2. Exercise Procedure

To induce muscle overuse, a laboratory model (a “kicking machine”) leading to marked overuse of the triceps surae muscle was used. Repetitive passive flexion and extension of the right ankle was achieved by means of a pneumatic piston and a band was placed around the hip/pelvis to restrict movements on the left side. During the plantar flexion phase, an active contraction was furthermore induced by electrical stimulation via surface electrodes (Pediatric electrodes 40 426A, Hewlett Packard, Andover, MA, USA) placed 2 cm apart over the triceps surae on the right side. The stimulation was synchronized with the plantar flexion movement of the piston by a microswitch, which trigged the stimulator unit (Disa stimulator Type 14E, Disa Elektronik A/S, Herlev, Denmark). A single impulse of 0.2 ms duration was delivered 85 ms after the initiation of the plantar flexion at an amplitude of 35–50 V. The stimulation intensity, which was tested out before the experiment, was submaximal and the intensity was individually corrected to obtain powerful muscle contractions. The movement frequency was 150 movements per minute. The left leg was not attached to the kicking machine. This exercise session lasted for 2 hours and was repeated every second day for 1, 3, or 6 weeks. The frequency and duration of repetitive movements were chosen in order to make a very marked strain on the muscles. The experimental procedures conform to those previously described [14, 15, 20] and do, with some amendments being made, conform to those described in studies by Backman and collaborators [21].

All animals were anaesthetized during the exercise procedure, by means of an intramuscular injection of fentanylfluanison (0.2-0.3 mL/kg) and diazepam (0.2 mL/kg; 5 mg/mL), followed by additional injections of fentanylfluanison (0.1 mL/kg) every 30–45 min during the experimental procedure in order to maintain anesthesia. For analgesia, buprenorphine, 0.01–0.05 mg/kg, was given s.c. postoperatively after each experiment session.

### 2.3. Collection, Freezing, and Sectioning of Muscle Samples

On the day after the last exercise, the rabbits were sacrificed by an intraperitoneal injection of an overdose of Pentobarbital natrium and the triceps surae with attached Achilles tendon was dissected out. The muscle samples were directly transported on ice to the laboratory. Further dissection was made, whereby samples conforming to the distal parts of the soleus and gastrocnemius muscles (5 × 8–10 mm) were further processed. These samples were fixed by immersion overnight at 4°C in an ice-cold solution of 4% formaldehyde in 0.1 M phosphate buffer (pH 7.0). They were thereafter thoroughly washed in Tyrode's solution containing 10% sucrose at 4°C overnight. The samples were mounted on thin cardboard in OCT embedding medium (Miles Laboratories, Naperville, IL, USA), frozen in propane chilled with liquid nitrogen, and stored at −80°C. Series of 7-8 *μ*m thin sections were cut using a cryostat. The sections were mounted on slides precoated with chrome-alum gelatine and were then processed for immunohistochemistry or morphology. Other series were sectioned for the purpose of being used for in situ hybridization. Additional samples were processed without being chemically fixed. These were directly mounted and frozen in the way described above and were sectioned and processed for the demonstration of tissue morphology.

### 2.4. Staining for the Demonstration of Morphology (Haematoxylin-Eosin)

Sections in the series were stained in Harris Haematoxylin solution for 2 min. They were then rinsed in distilled water, dipped in 0.1% acetic acid for a few seconds, followed by washing in running water. Counterstaining was achieved by immersion in eosin for 1 min. The sections were dehydrated in ethanol and mounted in Permount.

### 2.5. Immunohistochemistry

#### 2.5.1. Staining Procedures for the Demonstration of NK-1R

After washing with PBS 5 min × 3, incubation for 20 min in a 1% solution of Triton X-100 (Kebo lab, Stockholm, Sweden) in 0.01 M phosphate buffer saline (PBS), pH 7.2, containing 0,1% sodium azide as preservative, was performed, whereafter followed rinsing in PBS three times, 5 min each time. The sections were then incubated in 5% normal donkey serum (code no: 017-000-121, Jackson Immune Research Lab. Inc.) in PBS and were thereafter incubated with the NK-1R primary antibody, diluted in PBS (pH 7.4), in a humid environment. Incubation was performed for 60 min at 37°C. After incubation with specific antiserum and three 5-minute washes in PBS, another incubation in normal donkey serum followed, after which the sections were incubated with FITC-conjugated AffiniPure donkey anti-goat IgG (705-095-147; Jackson ImmunoResearch Lab Inc, dilution 1 : 100) for 30 min at 37°. The sections were thereafter washed in PBS and then mounted in Vectashield Mounting Medium (H-1000) (Vector Laboratories, Burlingame, CA, USA). Examination was carried out in a Zeiss Axioscope 2 plus microscope equipped with an Olympus DP70 digital camera.

As an alternative procedure, sections were initially pretreated with acid potassium permanganate for 2 min, a procedure found to enhance specific immunofluorescence reactions [22]. After this pretreatment, the procedures described above followed. Both procedures (with or without acid potassium treatment) gave in principle similar results. There were, however, some differences in staining intensities and levels of background reactions. It was therefore found useful to analyse sections by using both procedures.

#### 2.5.2. Double-Staining NK-1R Antibody/Tachykinin (SP) Antibody

The initial procedures conformed to the procedures described above concerning the staining for the NK-1R antibody. That included the use of FITC-conjugated AffiniPure donkey anti-goat IgG (705-095-147). The sections were then rinsed in PBS 4 × 2.5 min and incubated in 5% normal donkey serum in PBS for 15 min. The sections were thereafter incubated with a monoclonal tachykinin (SP) antibody (8450-0505, Biogenesis, Poole, UK), diluted 1 : 50 in PBS with BSA, for 60 min at 37°C. After incubation with this antiserum and 4 × 2.5-minute washes in PBS, a new incubation in normal donkey serum followed, after which the sections were incubated in TRITC-conjugated AffiniPure donkey anti-rat lgG (712-025-150) (Jackson ImmunoResearch, West Grove, PA, USA), diluted 1 : 40 in PBS supplemented with 0.1% BSA, for 30 min at 37°C. The procedures for washing and mounting were as described above. The reactions obtained with the tachykinin antibody are further on referred to as SP immunoreactive.

#### 2.5.3. Double-Staining NK-1R Antibody/Mouse Monoclonals

The initial procedures conformed also in this case to the procedures described above concerning the staining for the NK-1R antibody. That included the use of FITC-conjugated AffiniPure donkey anti-goat IgG (705-095-147) and rinsing in PBS 4 × 2.5 min, and as normal serum, 5% normal donkey serum in PBS was used. After the procedures for NK-1R immunolabelling were finished, the sections were rinsed in PBS 4 × 2.5 min and incubated in 5% normal rabbit serum in PBS with BSA for 15 min. After that, the sections were incubated with the other primary antibody to be stained for (all of which were mouse monoclonal antibodies) and which was against white blood cell markers, betaIII-tubulin, S-100beta, or desmin (see further below), and diluted in PBS with BSA, in a humid environment. Incubation was performed for 60 min at 37°C. After incubation with this antiserum and 4 × 2.5-minute washes in PBS, a new incubation in normal rabbit serum followed, after which the sections were incubated in rabbit anti-mouse immunoglobulins/TRITC (R0276) (Dako, Denmark). As an alternative procedure, normal donkey serum was used instead of rabbit normal serum, and in these cases, donkey anti-mouse immunoglobulins/TRITC (715-295-150) (Jackson ImmunoResearch) were utilized. Both variants gave similar and reliable results. The secondary antibody was used at a dilution of 1 : 40 (R0276) or 1 : 500 (715-295-150); the incubation with secondary antiserum in both cases proceeded for 30 min at 37°C. The sections were thereafter washed in PBS for 4 × 2.5 min and were then mounted in Vectashield Mounting Medium (H-1000) or Mounting Medium with DAPI (H-1500) (Vector Laboratories, Burlingame, CA, USA) in order to identify nuclei.

#### 2.5.4. Antibodies

An NK-1R antibody produced in goats was used (sc-5220, Santa Cruz). It is an affinity purified polyclonal antibody raised against a peptide mapping within an internal region of NK-1R of human origin. It was regularly used at a dilution of 1 : 50–1 : 100 in 0.1% in PBS. The tachykinin antibody used in double stainings was an SP monoclonal antibody produced in rats (8450–0505, Biogenesis, Poole, UK). It was used at a dilution of 1 : 50 in 0.1% BSA in PBS. The antibody 8450–0505 recognizes the COOH terminal end of SP and has been previously used for the immunohistochemical detection of SP in experimental animals and man [23].

The other antibodies used in double stainings were mouse monoclonal antibodies. One of these conformed to an antibody against CD68, (code no: M0814), from DAKOCytomation (Glostrup, Denmark), which was used at a dilution of 1 : 100 in 0.1% BSA in PBS. The antigen for this antibody is a glycosylated transmembrane glycoprotein, which is mainly located in lysosomes. Another one was an anti-rabbit T-cell/neutrophil antibody from AbD Serotec (Oxford, UK) (MCA805G), which was utilized at a dilution of 1 : 100 in 0.1% BSA in PBS. This antibody is an affinity purified antibody against a cell surface antigen, which is reported to be expressed by a subset of T cells, thymocytes, neutrophils, and platelets. A third antibody used was the antibody MAB1087 from Chemicon (Temecula, CA, USA), which reacts with human eosinophil peroxidase. This antibody was used at a dilution of 1 : 100 in 0.1% BSA in PBS. Furthermore, an antibody against betaIII-tubulin (T8660, Sima Aldrich, USA, 1 : 100) was used, being utilized at a dilution of 1 : 500. The antibody specificially recognizes an epitope located on isotype III of beta-tubulin. An antibody against S-100 (beta-subunit) was also utilized (S 2532) (Sigma, New York, NY, USA). It was used at a dilution of 1 : 500. The antibody recognizes an epitope located on the beta-chain, but not the alpha-chain, of S-100. An antibody against desmin was used as well (M0760). It was used at a dilution of 1 : 100. It is by the supplier (DAKOCytomation, Glostrup, Denmark) reported to give specific reactivity with desmin in a wide variety of tissues as seen via Western blotting experiments. It is furthermore reported not to show reactivity with other types of intermediate filaments [24]. Further descriptions of the antibodies are described elsewhere [16, 25, 26]. 

#### 2.5.5. Control Stainings

Control stainings concerning the NK-1R antibody included the use of NK-1R blocking substance (sc-5220P) (50 *μ*g/mL antiserum). Ordinary stainings for NK-1R were made in parallel. Concerning controls for the SP antibody, blocking with SP peptide (full length SP peptide) from Sigma (S6883) (50 *μ*g/mL antiserum) was used. Other control stainings conformed to stainings when the primary antibodies were excluded (buffer instead of the antibody). The characteristics of antibodies used have also been evaluated in previous studies [23–25].

### 2.6. In Situ Hybridization (ISH)

In order to complement the results obtained at the protein level (via immunohistochemistry), stainings to show the situation at the mRNA level (via in situ hybridization) were performed. Digoxigenin- (DIG-) hyperlabelled oligonucleotide triple probe cocktail (ssDNA) for the detection of the NK-1R, also known as tachykinin receptor 1 (TACR1) (Gene Detect, New Zealand), was thus used. A “triple probe cocktail” was prepared as the exact rabbit TACR1 sequence is not yet cloned. This contained three antisense probes directed towards both human and rat TACR1 mRNA, which is considered to have a very high probability of detecting rabbit TACR1 mRNA. The antisense probe sequences were

- probe #1: GGCTGCACGAACTGGTTAGACTCAGAGGTGTTGGTGGAGATGTTGGGG,
- probe #2: TGGAGCTTTCTGTCATGGTCTTGGAGTTGCTGCGAGAGGAGCCGTTGG,
- probe #3: TGACCACCTTGCGCTTGGCAGAGACTTGCTCGTGGTAGCGGTCAGAGG.

As a negative control, a “triple probe cocktail” of the corresponding sense DIG-hyperlabelled ssDNA probes was used. Also *β*-actin antisense/sense probes (GD5000-OP) (GeneDetect, New Zealand) were used for control purposes.

Based on the features noted concerning tissue morphology (myositis affection) and NK-1R immunohistochemical reaction patterns, certain samples were chosen for the in situ hybridization. These corresponded to 1 sample from the control nonexercised group, 1 sample from the 1-week group (exercised side), and 2 samples from the 6-week group (one from nonexercised side and one from exercised side). Two of the samples were from the soleus muscle and two were from the gastrocnemius muscle. It was considered that the samples used were representative samples.

ISH was performed according to an established protocol [27] using an alkaline-phosphatase- (AP-) labelled anti-DIG antibody for detection, with a few modifications [28, 29]. The cryostat knife was washed in 70% EtOH in diethyl pyrocarbonate [DEPC]–H2O and the sections were mounted on Super Frost Plus slides (nr. 041300, Menzel-Gläser, Braunschweig, Germany). Concerning the further procedures, see [28, 29]. In brief, an aliquot (50 ng) of the ssDNA probe was added to 15 *μ*L of hybridisation solution in a 1.5 mL microfuge tube, denatured for 5 min in 80°C and then put on ice. The hybridisation solution was as follows: 500 *μ*L formamide, 200 *μ*L 20x SSC, 50 *μ*L of 20x Denhardt's solution, 50 *μ*L herring sperm DNA [10 mg/mL] heat denatured, 25 *μ*L baker's yeast RNA [10 mg/mL], 175 *μ*L dextran sulphate [50%], and total volume was: 1.0 mL. The probe-containing hybridisation solution was then applied to each section, the sections being covered with cover slips and sealed with nail polish. Incubation followed at 56°C overnight.

### 2.7. EIA Method

In order to complement the NK-1R reaction studies, EIA analysis of SP content for the muscle specimens was made. For this purpose, muscle samples were frozen in liquid nitrogen directly after being weighed, whereafter they were homogenized, by the use of Precellys 24 tissue homogenizer (Bertin Technologies, Saint Quentin en Yvelines, Cedex, France), and further processed for EIA using commercially available enzyme immunoassay SP kit (Phoenix Pharmaceuticals, Burlingame, CA, USA). For further details of the procedures, see [30]. Analysis was restricted to the analysis of control muscles and muscles of 6-week groups. The assay was conducted in accordance with the instructions from the manufacturer. An SP EIA analysis evaluating all the various groups will be conducted and described in a forthcoming study.

### 2.8. Statistics

Independent T-tests were done concerning evaluations of differences in SP concentration between controls and 6w groups. The statistical software SPSS PASW Statistics 18 (SPSS Inc, Chicago, USA) was used. A *P*-value < 0.05 was considered to be significant.

### 2.9. Ethics

The study protocol was approved by the local ethical committee at Umeå University (A 34/07). The approval was obtained before the start of the study. A licensed breeder had bred all animals for the sole purpose of being used in animal experiments. All efforts were made to minimize animal suffering.

## 3. Results

### 3.1. Morphology

**Figure 1:**
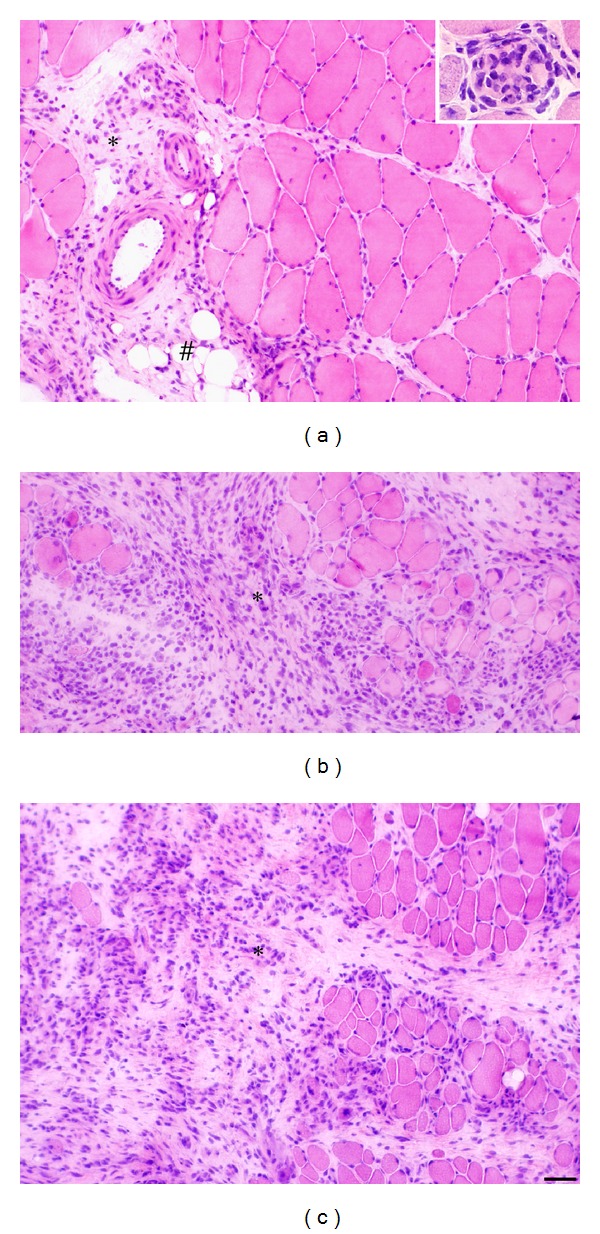
Sections of the soleus muscle of the 6-week group, exercised side (a, b) and nonexercised side (c), stained with H&E. Morphological features are shown. There is an excess of connective tissue (asterisk) and presence of adipose tissue to the left in (a). There are pronounced inflammatory cell infiltrates in (b, c) (asterisks). There is an overall marked variability in muscle fiber sizes (b, c). In the inset in (a), a fiber that is invaded by numerous inflammatory cells is seen. The appearance to the right in (a) is to some extent resembling the situation for normal muscle. (#) indicates adipose tissue. Bar = 50 *μ*m.

The specimens of the soleus and gastrocnemius muscles showed marked morphological changes in response to the experimental procedure. The changes were especially pronounced after the 6-week experiment period (Figure 1). However, morphological alterations were also to some extent seen in the 1-week and 3-week groups. The morphological changes included an occurrence of marked inflammatory infiltrates, a pronounced variability in muscle fiber size, and occurrence of marked muscle fiber alterations (morphologically “abnormal muscle fibers”) (Figure 1). Numerous white blood cells could be seen to be dispersed in the tissue (Figures 1(b) and 1(c)). The morphological changes were seen in certain parts of the specimens. In other areas of the specimens, the morphology was seemingly unaffected.

Importantly, morphological changes occurred to an almost similar extent in the muscles in the contralateral nonexercised side as in the muscles in the exercised side (Figures 1(b) and 1(c)). The pattern for the morphological changes was the same for the soleus and the gastrocnemius muscles, but the extent of the changes was somewhat more pronounced for the soleus muscle. The findings concerning the morphology are in accordance with previous findings for the triceps surae muscle in response to the experimental regimen here used [14].

### 3.2. Immunohistochemistry

#### 3.2.1. Reactions in the Invading White Blood Cells

**Figure 2:**
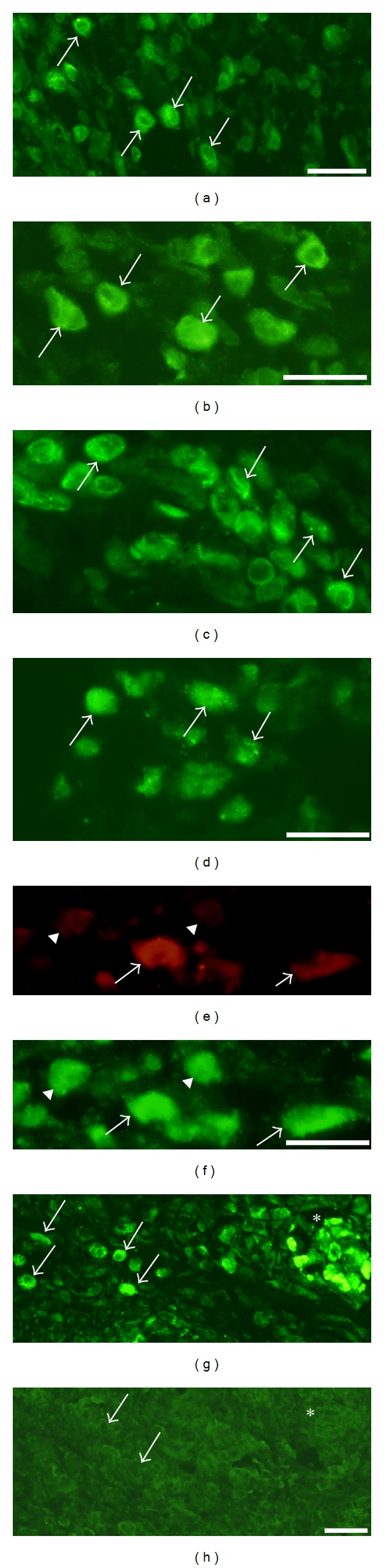
Immunoreaction patterns for cells in inflammatory infiltrates are shown. Sections are from gastrocnemius muscle specimens, 1-week group ((a), (b), and (e)–(h)), 6-week group (c), exercised sides, and soleus muscle specimen of the 6-week group, nonexercised side (d). In (a)–(d), the presence of cellular NK-1R immunoreactions is seen (arrows). The reactions show frequently a granular appearance. Double staining for SP (e) and NK-1R (f) show that there is partly colocalization (arrows), partly not colocalization (arrowheads). Ordinary staining for NK-1R shows the presence of marked immunoreactions in cells (g) (arrows) (low magnification), whereas there are no specific immunoreactions in a parallel section processed for NK-1R after preabsorption with NK-1R antigen (h). The immunoreactive cells seen in (g) are scattered in the connective tissue and coalesced into a muscle fibre (asterisk (g), c.f. (h)). Arrows in (h) point at nonimmunoreactive cells. Bars = 25 *μ*m.

**Figure 3:**
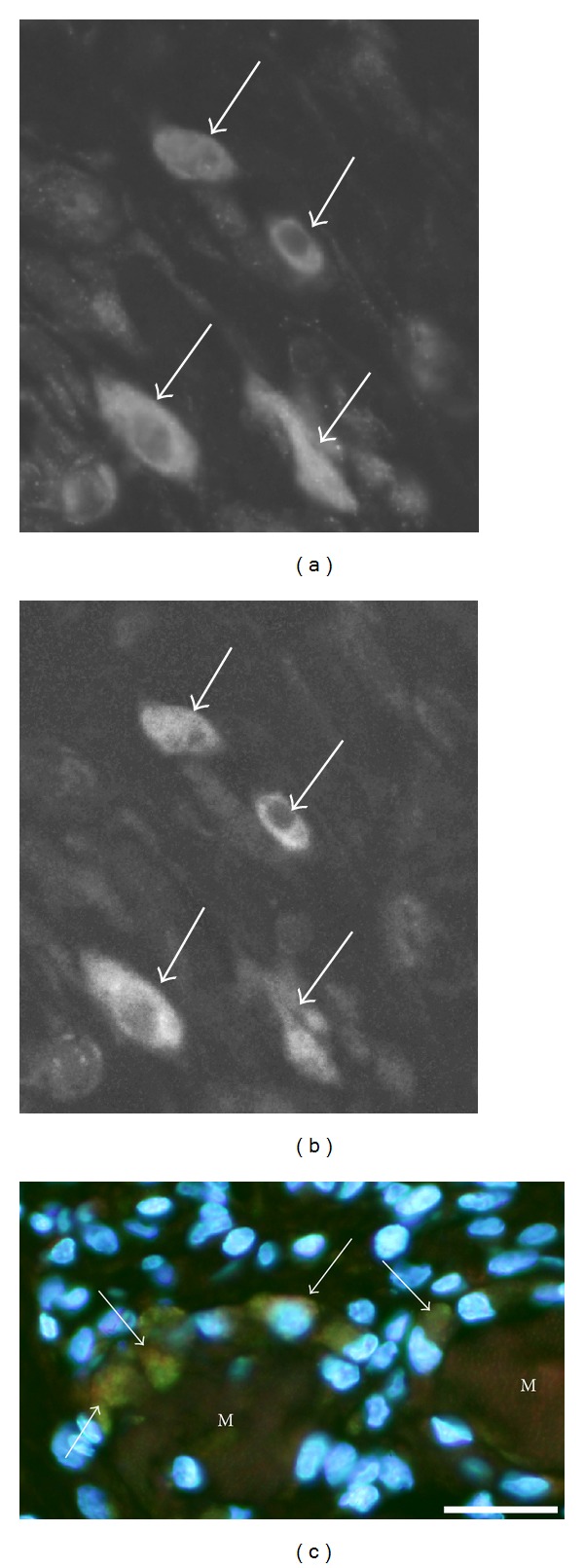
Results of double stainings for cells of inflammatory infiltrates. Sections are from gastrocnemius muscle specimen of the 1-week group, exercised side. Double staining for NK-1R (a) and CD68 (b). There is colocalization in cells (arrows). Double staining for NK-1R (green) and eosinophil marker (MAB1087) (reddish) is shown in a single montage (DAPI in mounting medium) in (c). There is colocalization in eosinophils (arrows). M = muscle fibers. Bar = 25 *μ*m.

Immunoreactions for NK-1R occurred in cells that were dispersed in the inflammatory infiltrates (Figure 2). That was the situation for both the exercised (Figures 2(a)–2(c)) and nonexercised (Figure 2(d)) sides and for both muscles. The reactions frequently showed a granular/punctuate appearance. Double stainings showed that parts of the NK-1R immunoreactive cells were also SP immunoreactive, whilst others did not show SP immunoreaction (Figures 2(e) and 2(f)). The NK-1R immunoreactions were abolished in sections processed with NK-1R antiserum that had been preabsorbed with NK-1R antigen (Figures 2(g) and 2(h)). The NK-1R immunoreactive cells were found to correspond to CD68 immunoreactive cells, that is, macrophages (Figures 3(a) and 3(b)), or eosinophils (Figure 3(c)). There were no NK-1R immunoreactions in cells showing reactions for the antibody that was a T-cell/neutrophil marker.

#### 3.2.2. Reactions in Muscle Fibers Showing Infiltration of White Blood Cells

**Figure 4:**
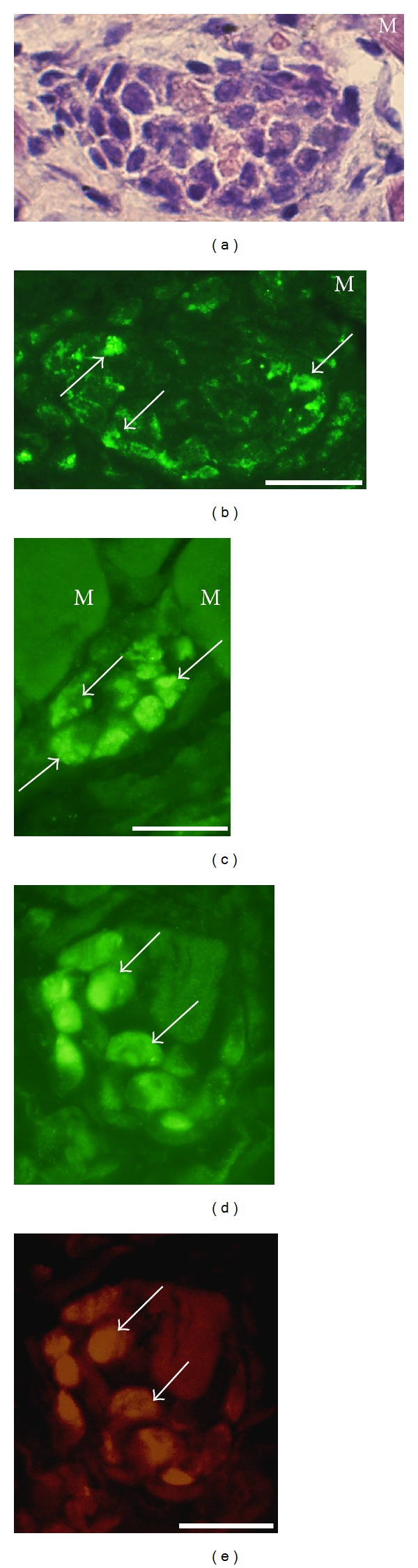
Sections of soleus muscle specimens from the 6-week group, nonexercised side (a, b) and the 1-week group, exercised side (c), and of gastrocnemius muscle specimen from the 1 week group, exercised side (d, e). Abnormal muscle fibers, that is, muscle fibers being completely infiltrated by white blood cells, are shown. Such a fiber is seen in the middle in (a) (H&E staining). There are cellular NK-1R immunoreactions in this fiber (b) (arrows). There are also cellular NK-1R immunoreactions in (c) and (d) (arrows indicate immunoreactive cells). (d) and (e) represent double staining for NK-1R (d) and SP (e). There is colocalization (arrows). M = ordinary muscle fibers. Bars = 25 *μ*m.

White blood cells were not only seen to be dispersed in the tissue, but could also be seen to be coalesced into muscle fibers (morphologically “abnormal muscle fibers”) (c.f. above) in the myositis areas (Figure 1(a)). The stainings for NK-1R showed that there were frequently NK-1R immunoreactions in the cells that had invaded the muscle fibers (Figures 4(a)–4(d)). That was the situation for exercised as well as nonexercised sides and for soleus as well as gastrocnemius muscles. As was the case for the white blood cells that were dispersed in the tissue, white blood cells within the muscle fibers were also immunolabelled for eosinophil marker or CD68. Double-staining NK-1R/SP showed that SP immunoreactions were frequently detected in the NK-1R immunoreactive cells within the muscle fibers (Figures 4(d) and 4(e)). The characteristics of these abnormal muscle fibers are further clarified below.

#### 3.2.3. Reactions in Nerve Structures

**Figure 5:**
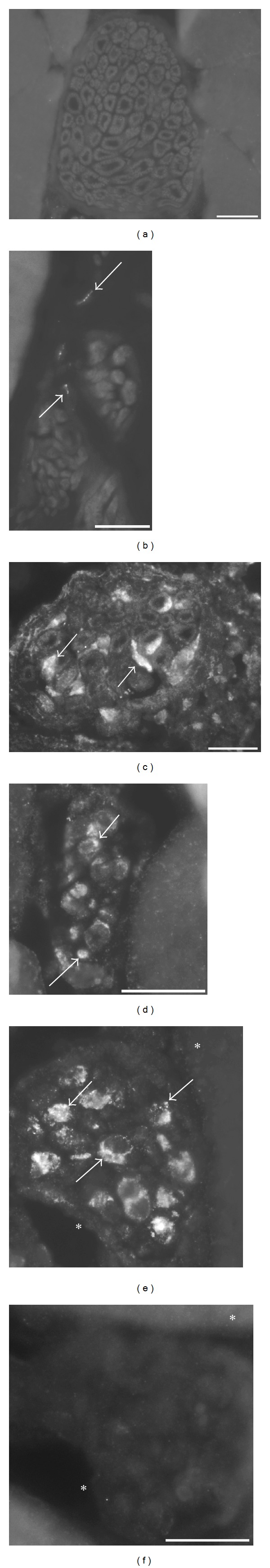
NK-1R immunoreaction patterns in nerve fascicles. Sections of soleus muscle specimens from the control group (a), the 1-week group, exercised side (b) and the 6-week group, nonexercised side (c) and of gastrocnemius muscle specimens from the 6-week group, exercised side (d) and nonexercised side (e, f). The nerve fascicle shown in (b) was located in a region showing normal muscle morphology whilst those shown in (c)–(e) were localized to myositis areas. Note that there are no NK-1R immunoreactions in the nerve fascicle in (a). This fascicle is seemingly entirely composed of myelinated nerve fibers. There are also no immunoreactions in the nerve fascicles in (b) (above and below). Just outside these, there are fine varicose immunoreactive nerve fibers (arrows). On the other hand, there are marked NK-1R immunoreactions in the nerve fascicles shown in (c)–(e) (arrows). These show a punctuate appearance, with the reactions to some extent, however, being spread out in broad areas. In (f), a section parallel to the section shown in (e) is seen, and for which the NK-1R antibody had been preabsorbed with NK-1R peptide. There are no NK-1R immunoreactions in the nerve fascicle in (f). Asterisks mark corresponding regions in (e) and (f). Bars = 25 *μ*m.

Nerve fascicles of various dimensions were seen in specimens of both muscles. Based on the morphologic appearance, a large number of these were entirely composed of myelinated nerve fibers. Immunohistochemical analysis revealed that NK-1R immunoreactions were never observed within nerve fascicles being entirely built up by myelinated nerve fibers (Figure 5(a)).

On the whole, the nerve fascicles of control samples and those located in normally appearing muscle areas of experimental animals were nonimmunoreactive for NK-1R (Figures 5(a) and 5(b)). Occasional immunoreactive nerve fibers with a varicose appearance were, however, noted close to these nonimmunoreactive nerve fascicles (Figure 5(b)). Such nerve fibers could also be observed in association with blood vessels in connective tissue spaces.

**Figure 6:**
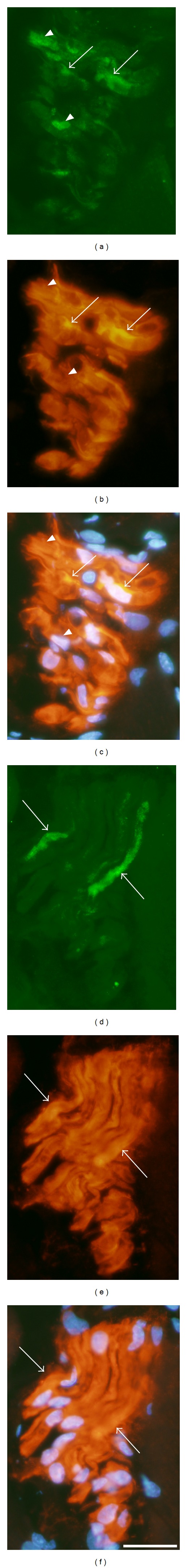
Double staining for NK-1R (a, d) and S100 (*β*-subunit) (b, e). Merged images including DAPI reactions are shown in (c, f). Large nerve fascicles are seen. Sections are from gastrocnemius muscle specimen of the 6-week group, nonexercised side. There is NK-1R immunoreaction and to some extent an extra marked S-100beta immunoreaction in some regions (arrows) (a)–(f). For some regions there is NK-1R immunoreaction but not this type of marked S-100beta immunoreaction (arrowheads). Note that the nuclei within the nerve fascicles show a pink colour reaction whilst those located outside these show the characteristic blue DAPI reaction in (c, f). This means that the nuclei located inside the nerve fascicle exhibit the blue DAPI reaction merged with the colour reaction from the S-100beta staining. Bar = 25 *μ*m.

Marked immunoreactions for NK-1R were, on the other hand, noted in nerve fascicles of large/considerable sizes that were located in myositis areas and in the close proximities of these areas for both muscles and for both exercised and nonexercised sides. The reactions were point-like but were frequently coalesced. Due to the occurrence of marked reaction intensities for these, broad areas were partly seen to exhibit fluorescence (Figures 5(c)–5(e)). These types of immunoreactions were especially seen in the 6-week groups (Figures 5(c)–5(e)) but could also be seen in myositis areas of the 1- and 3-week groups. Such immunoreactions for nerve fascicles were never seen in areas of muscles from experimental animals showing normal morphology and were never seen in the specimens of the control nonexperimental animals (c.f. above). Double stainings revealed that these NK-1R immunoreactions frequently, but not always, were associated with comparatively marked S100beta immunoreactions (Figure 6).

The nuclei of the nerve fascicles in which the NK-1R immunoreactions described above were noted and which thus were located in myositis areas showed, when DAPI was used in embedding medium and when S100beta immunolabelling was performed, another colour reaction than the nuclei located outside the nerve fascicles (Figures 6(c) and 6(f)). The former nuclei showed a pink colour reaction, whilst the latter exhibited a bluish reaction; that is, a colour that is characteristic of the DAPI reaction. The nuclei of nerve fascicles located in normally appearing muscle tissue mainly showed the characteristic bluish DAPI reaction.

**Figure 7:**
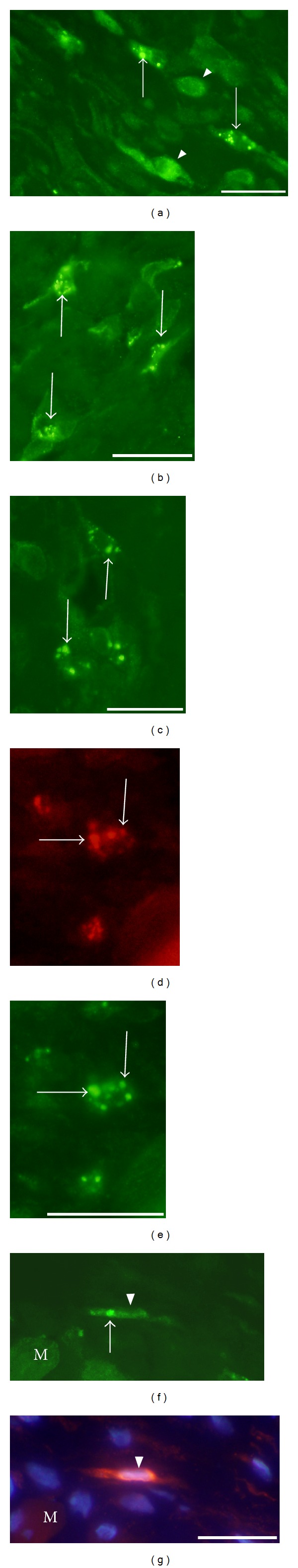
Sections stained for the demonstration of NK-1R (a)–(c). In (d, e), double-staining for SP (d) and NK-1R (e) is shown. In (f, g), double staining for NK-1R (f) and S100beta (g) is shown. Specimens are from gastrocnemius muscle, exercised side, 1-week group (a, d, e), gastrocnemius muscle, nonexercised side, 6-week group (b, f, g), and soleus muscle, exercised side, 6-week group (c). In (a), strong NK-1R immunoreactions are seen in nerve fibers (arrows) and weaker immunoreactions in inflammatory cells (arrowheads). In (b, c), immunoreactive nerve profile reactions are seen (arrows). In (d, e) it is obvious that there is only partial colocalisation between SP- and NK-1R immunoreactions in the nerve fibers (arrows). In (f), coalesced NK-1R immunoreactive profiles are seen. These are related to a markedly S100beta-immunoreactive cell (arrowhead at nucleus). Note that this nucleus exhibits a pink colour reaction; other nuclei in (g) show the characteristic bluish DAPI reaction. M= muscle fiber (f, g). Bars = 25 *μ*m.

**Figure 8:**
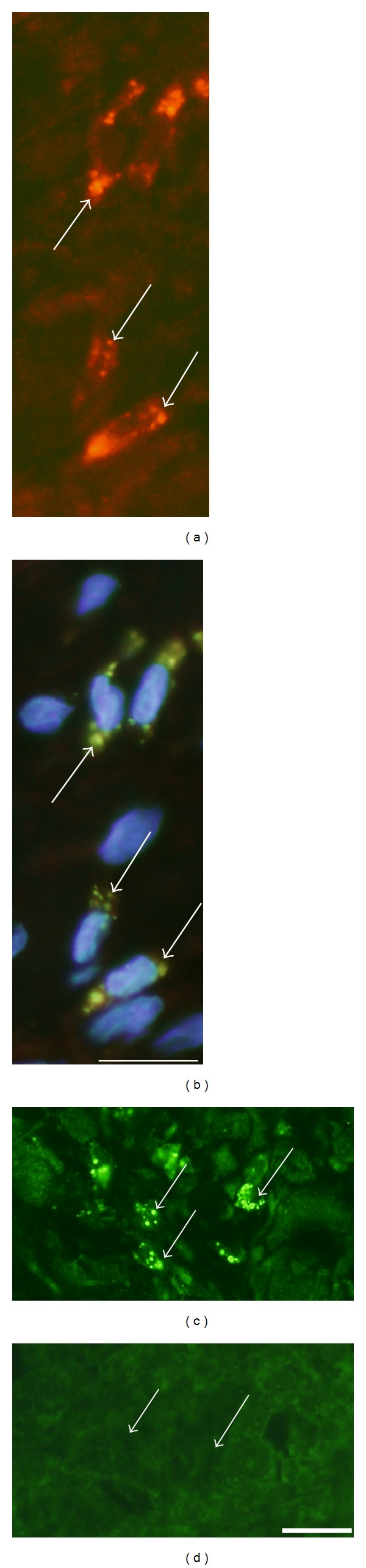
Sections from gastrocnemius muscle of 1-week exercised group. Double staining for NK-1R and *β*-Tubulin is shown in (a, b). *β*-Tubulin immunoreactions ((a), red, arrows) show colocalization with NK-1R immunoreactions ((b), green, arrows). In (b), the DAPI reaction is also seen. Results of preabsorption stainings are shown in (c, d), ordinary staining for NK-1R in (c), and preabsorption staining in (d). The region in (c) corresponds to that in (d). There are axonal NK-1R immunoreactions in (c) (arrows). There are no NK-1R immunoreactions in the preabsorption control (d). Bars = 25 *μ*m.

Via parallel double stainings for betaIII-tubulin/NK-1R and S100beta/NK-1R and via comparisons with parallel stainings made for white blood cells further information on nerve-related NK-1R reactions was obtained. Strong NK-1R immunoreactions that were not present in large nerve fascicles and that were not inflammatory cell related were thus seen in myositis areas of both sides and in close proximities to these areas (Figures 7(a)–7(c)). These strong NK-1R immunoreactions were betaIII-tubulin immunoreactive (Figures 8(a) and 8(b)). It was hereby verified that these corresponded to axonal profiles. The NK-1R immunoreactions were associated with strong S100beta immunoreactions (Figures 7(f) and 7(g)). Parts of the NK-1R immunoreactive axonal profiles were SP immunoreactive (Figures 7(d) and 7(e)). These axonal NK-1R immunoreactions (Figures 8(c) and 8(d)), as well as the point-like NK-1R immunoreactions seen in large nerve fascicles (Figures 5(e) and 5(f)), were abrogated by preabsorption with NK-1R antigen.

#### 3.2.4. Reactions in Blood Vessel Walls and Fibroblasts

**Figure 9:**
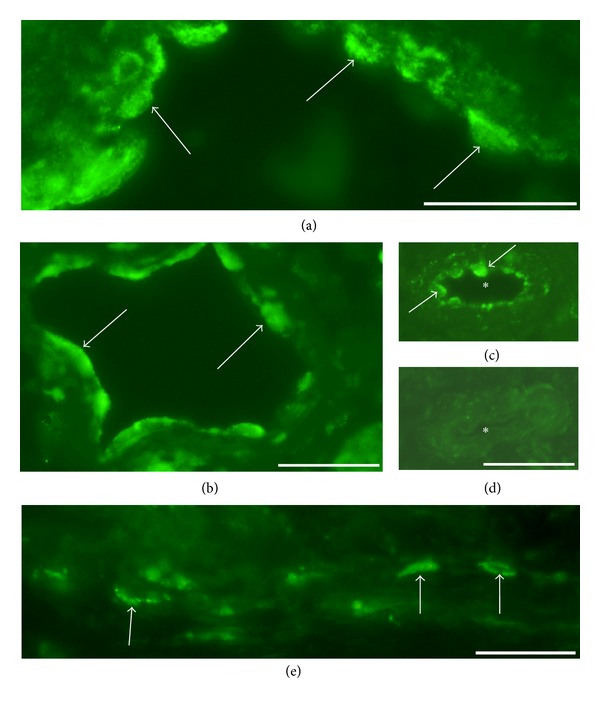
(a)–(d) NK-1R immunoreactions in the walls of blood vessels located in myositis areas or close to these areas are shown (preabsorption control in (d)). Sections are from gastrocnemius muscles from the exercised side, the 6-week (a) and 1-week (b) groups, and from the nonexercised side, 3-week group (c, d). Note that there are NK-1R immunoreactions in the blood vessel walls in both exercised and nonexercised sides (arrows, (a)–(c)). The reactions are observed in the endothelial layer of the vessels. There is no reaction in the preabsorption control (d). The vessel shown in (d) conforms to that in (c) but was sectioned in another level of the sectioning, why the vessel morphology is different. Asterisks in the lumen of the blood vessel (c, d). Bars = 25 *μ*m. (e) Immunostaining for NK-1R. Soleus muscle specimen of the 6-week group, nonexercised side. There are immunoreactions in fibroblast-like cells (arrows). Bar = 25 *μ*m.

NK-1R immunoreactions were noted for the endothelial layer of some of the blood vessels in myositis areas and areas located in the close proximities of these areas (Figure 9). They were observable for small and intermediate-sized vessels. Blood vessel-related NK-1R immunoreactions were never seen for the walls of blood vessel walls of controls and those in normally appearing muscle tissue of experimental animals and were in principle not seen for the walls of large arteries of either of the groups. The reactions were abrogated via NK-1R preabsorption (Figures 9(c) and 9(d)). NK-1R immunoreactions were also seen for fibroblast-like cells located in connective tissue spaces (Figure 9(e)).

#### 3.2.5. NK-1R in Muscle Fibers in Relation to Desmin Immunoreaction

**Figure 10:**
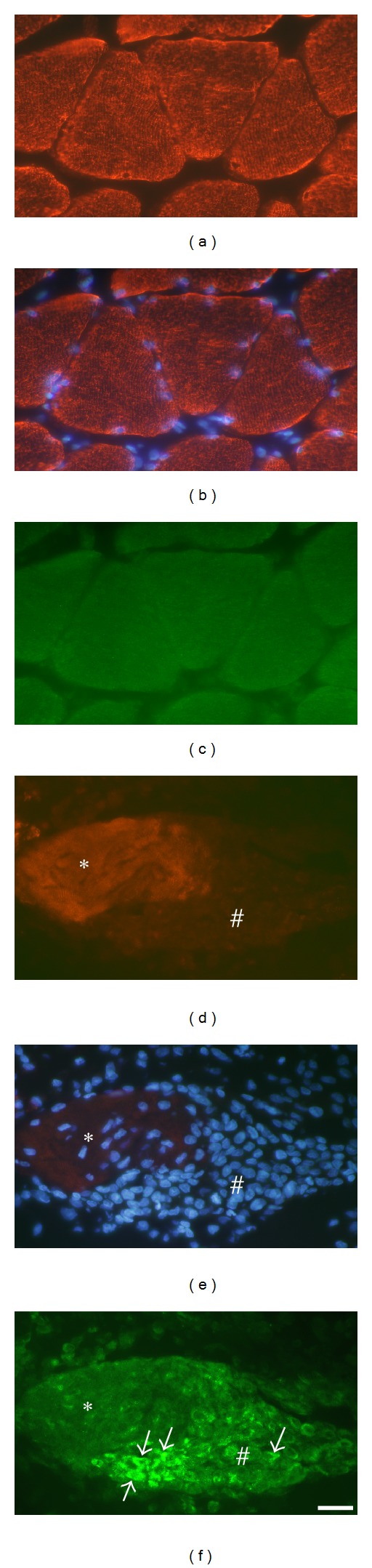
Parts of sections from a soleus muscle specimen, exercised side, 6-week group. In (a)–(c) a normally appearing muscle area is shown, in (d)–(f) a part of a myositis area. The muscle fibers in (a)–(c) are in principle transversely cut, whereas those in (d)–(f) are longitudinally cut. Double staining for desmin and NK-1R. Desmin staining is shown in (a, d), desmin staining coupled to DAPI in (b, e), and NK-1R staining in (c, f). In (a, b), the normal striated desmin immunoreaction pattern is seen. There are no NK-1R immunoreactions in (c). In (e), it is seen that there is a massive infiltration of cells in a part of the muscle fiber (#), and to a certain extent an infiltration in another part (asterisk). The desmin immunoreaction is weak in the middle of the latter part and non-existent in the former. There are NK-1R immunoreactions in infiltrating cells (arrows, (f)). Bar = 25 *μ*m.

**Figure 11:**
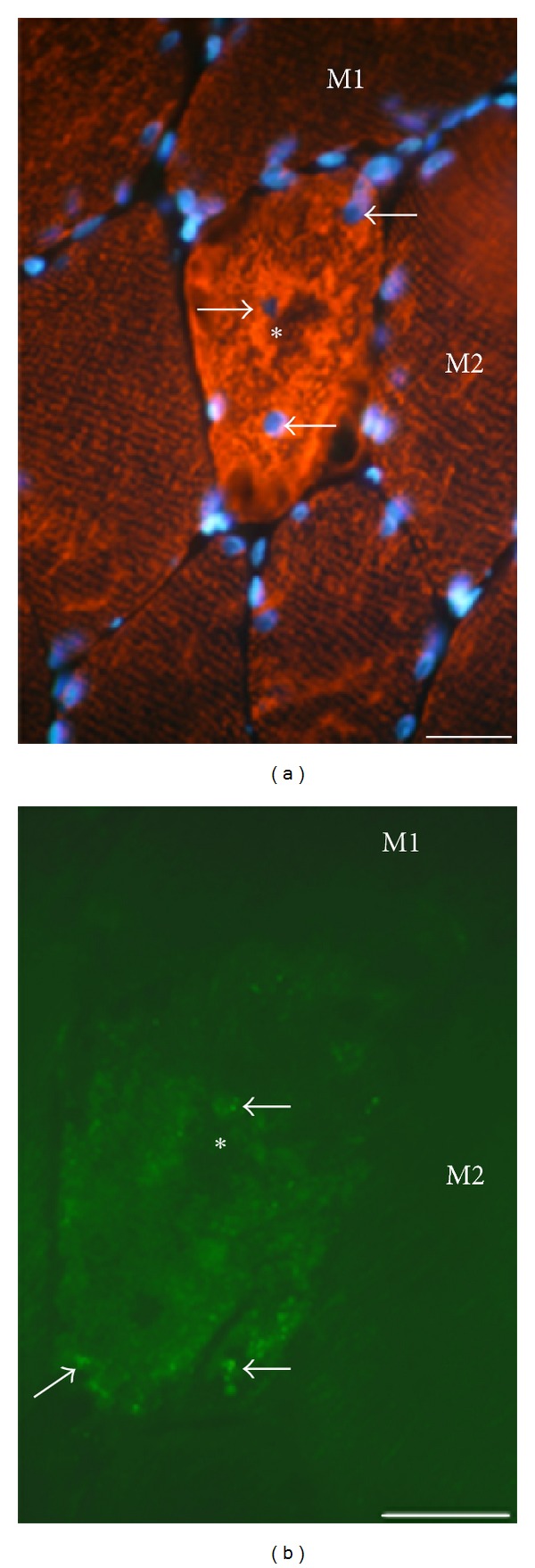
Section from a soleus muscle specimen, nonexercised side, 6-week group. Double staining for desmin coupled to DAPI (a) and NK-1R (b) is shown. The area is shown in higher magnification in (b) than in (a). There is a very marked desmin immunoreaction in the muscle fiber in the middle (asterisk), and the normal desmin striations cannot be seen in the fiber. These striations can be seen in adjacent muscle fibers. There are point-like NK-1R immunoreactions in this muscle fiber (arrows, (b)) but no NK-1R in adjacent fibers. M1, M2 = muscle fibers. There are internal nuclei in the muscle fiber in the markedly desmin immunoreactive muscle fiber (arrows, (a)). Bar = 25 *μ*m.

In order to further clarify the NK-1R reactions in muscle fibers, double staining for desmin and NK-1R was performed. In muscle fibers that were heavily infiltrated by white blood cells (c.f. above) and for which NK-1R was frequently detected in these cells, there was a marked decrease or a lack of desmin immunoreactivity. The lack of desmin immunoreaction could be seen in parts of the muscle fiber (Figure 10) or in the entire muscle fiber. Muscle fibers with an increased desmin immunoreaction were also seen (Figure 11). These muscle fibers contained more internal nuclei than normally seen (Figure 11). In these fibers, there was also NK-1R immunoreaction, with the immunoreaction in this case being scattered in the form of punctuate reactions (Figure 11).

Both types of muscle fibers described above showing NK-1R immunoreactions and an aberrant desmin immunoreaction were observed in myositis areas or in areas that were closely adjacent to these areas. They were very occasionally seen in other areas of muscle samples of experimental animals and were never seen in muscle samples of control animals. Other muscle fibers in the myositis areas and in normally appearing muscle tissue of experimental animals and all muscle fibers in the specimens of control animals showed the characteristic desmin immunoreaction pattern, that is, a striated reaction pattern in longitudinally cut muscle fibers (Figure 11). These muscle fibers were NK-1R nonimmunoreactive (Figure 11).

#### 3.2.6. Comparisons Exercised versus Nonexercised Musdes Overall Comments

The pattern and magnitude of the NK-1R immunoreactions for all the various structures were similar for exercised and nonexercised sides. The marked NK-1R immunoreactions seen for white blood cells, nerve fascicles, blood vessel walls, and muscle fibers described above were restricted to myositis areas and areas that were very close to these areas. That was the case for both the soleus and gastrocnemius muscles. Thus, the magnitude of the NK-1R immunoreactions was related to the degree of inflammatory infiltrates (myositis) within the specimens, which to some extent varied between the different samples in the experimental groups [14].

### 3.3. In Situ Hybridization

**Figure 12:**
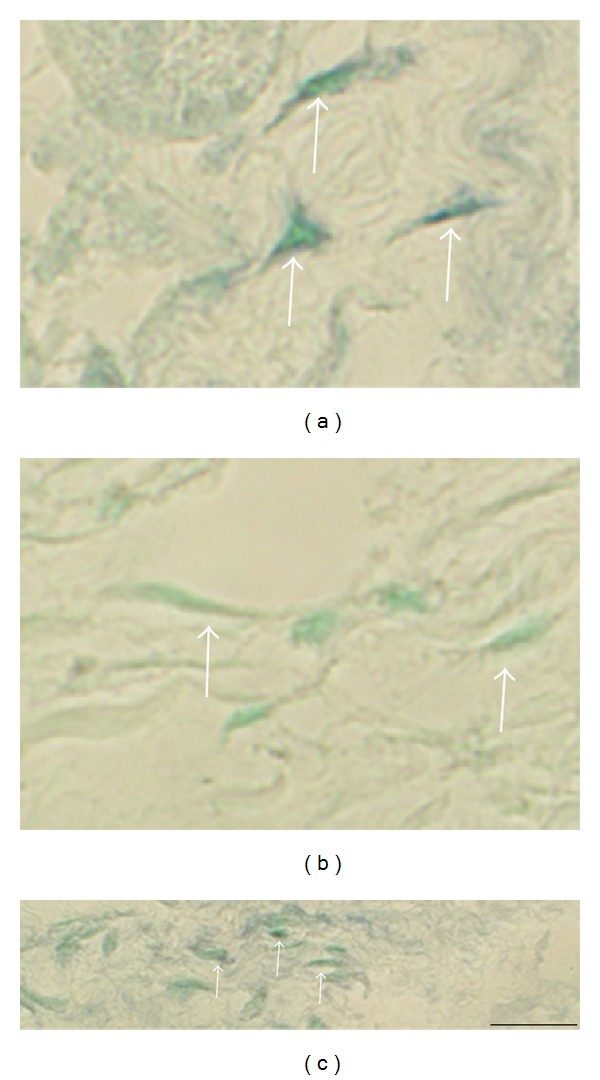
Reactions in fibroblast-like cells. Sections of a gastrocnemius muscle specimen of the 1-week group, exercised side (a, b), and a soleus muscle specimen of the 6-week group, exercised side (c), are shown. In situ hybridization (antisense staining in (a), (c); sense staining in (b); (b) is from a parallel section to (a)). There is an expression of NK-1R (TACR1) mRNA in the cells in (a). There are no reactions in the sense control (b). Arrows point at fibroblast-like cells. There are also NK-1R mRNA reactions in the fibroblast-like cells in (c) (arrows). Bar = 25 *μ*m.

**Figure 13:**
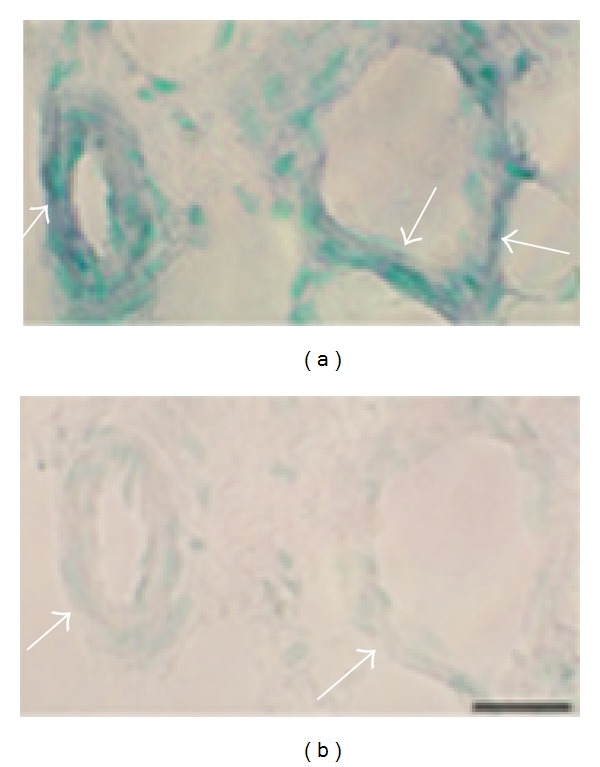
Expression of NK-1R (TACR1) mRNA in blood vessel walls. Serial sections from soleus muscle specimen of the 6 week group, non-exercised side, showing small arteriole (left part, (a), (b)) and vein (right part, (a), (b)). Processing for NK-1R mRNA using anti-sense probe (a) and processing with corresponding sense probe (b). Expression of NK-1R mRNA is seen in the walls of the blood vessels in (a). There are no reactions in the sense control (b). Arrows indicate parts of the walls. Bar = 25 *μ*m.

**Figure 14:**
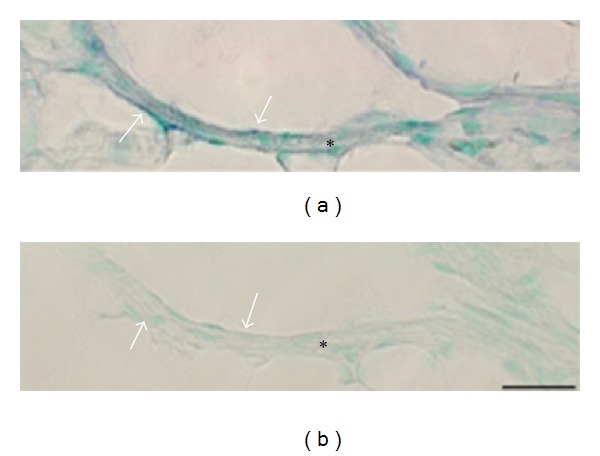
Expression of NK-1R (TACR1) mRNA in the wall of a large vein is shown (soleus muscle specimen of 6w group, nonexercised side). Processing for NK-1R mRNA using anti-sense probe (a) and processing with corresponding sense probe (b). Expression of NK-1R mRNA is seen in the vein wall (a). There are no reactions in the sense control (b). Arrows indicate parts of the wall. Asterisks indicate similar parts of the vessel wall. Bar = 25 *μ*m.

**Figure 15:**
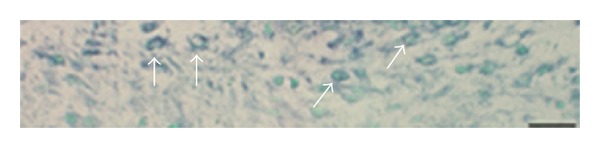
Expression of NK-1R (TACR1) mRNA in inflammatory infiltrate. Staining for the demonstration of NK-1R mRNA (antisense probe) in a section of a gastrocnemius muscle specimen, exercised side, 1-week group, is shown. Cells in an inflammatory infiltrate are showing NK-1R mRNA reactions (arrows). Bar = 25 *μ*m.

**Figure 16:**
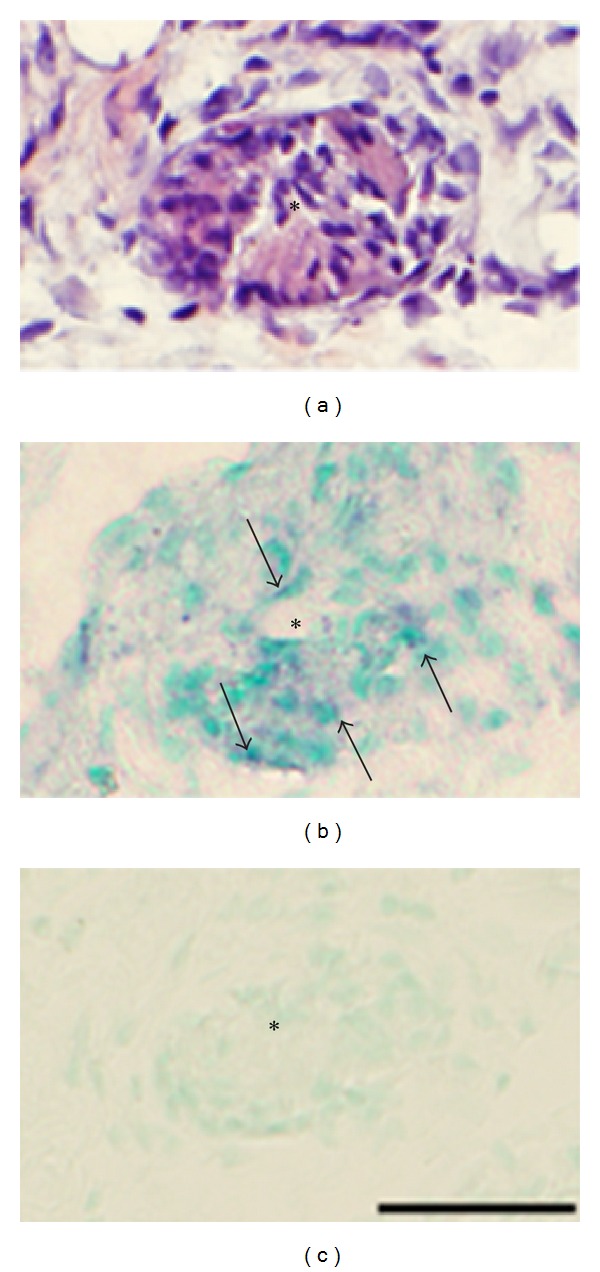
Expression of NK-1R (TACR1) mRNA. Serial sections of a soleus muscle specimen of the 6-week group, nonexercised side, showing an abnormal muscle fiber (asterisks). In (a), the fiber is shown to be markedly infiltrated by inflammatory cells (staining with H&E). There is NK-1R mRNA expression in this fiber (b) (arrows). No reactions are visible in the sense control (c). Bar = 25 *μ*m.

The in situ hybridization studies showed that NK-1R mRNA reactions were observable in the same cell structures as were found to be NK-1R immunoreactive. That included fibroblast-like cells (Figure 12), cells of blood vessel walls in myositis areas and/or closely adjacent areas (Figures 13 and 14), white blood cells in inflammatory infiltrates (Figure 15), and muscle fibers showing abnormal morphology, being heavily infiltrated by cells (Figure 16) or containing frequent internal nuclei. The NK-1R mRNA reactions in blood vessel walls were not only localized to the endothelial layer but could also be seen for the smooth muscle part of the walls (Figure 13). The specificity of reactions seen for all the various structures was verified via stainings with sense control probe (Figures 12(b), 13(b), 14(b), and 16(c)).

### 3.4. EIA Results

Analysis of SP concentration was made for muscles of control animals and animals for which the most pronounced myositis was observed, that is, the 6-week groups. It was found that the concentration was clearly higher in the 6-week groups for both muscles and for both sides. The mean values were thus 1.83 times higher for exercised side and 1.77 times higher for nonexercised side, as compared to control muscle, concerning the soleus muscle (*P* = 0.004 in both cases), and 3.45 times (exercised side) and 3.01 times (nonexercised side) higher than the mean value in the control group concerning the gastrocnemius muscle (*P* < 0.05 in both cases).

## 4. Discussion

### 4.1. Summary of Findings

Via evaluations of the NK-1R reaction pattern, the present study indicates that tachykinins are highly involved functionally in the myositis and muscle affection process in our rabbit model of muscle overuse. An upregulation of the expression of NK-1R was observed in both the exercised as well as the nonexercised sides. NK-1R immunoreactions and NK-1R mRNA reactions were seen for cells of the inflammatory infiltrates and for blood vessel walls within the inflamed and affected areas. Such reactions were also seen for fibroblasts. Strong NK-1R immunoreactions in axonal profiles and fine NK-1R immunoreactions in large-sized nerve fascicles were furthermore frequently detected in the areas influenced by the myositis process. Additionally, morphologically abnormal muscle fibers in these areas displayed NK-1R immunoreactions and NK-1R mRNA reactions. The NK-1R reactions were thus in principle restricted to the myositis areas and the areas located very close to these areas.

When interpreting the significance of the NK-1R immunoexpressions, one should be aware of the fact that not only SP but also endokinins and hemokinins show a remarkable potency for NK-1 receptors [31]. Nevertheless, in the tachykinin stainings performed in the present study antibodies against SP (monoclonal antibodies) were used.

### 4.2. Crossover Effects of the Tachykinin System

An interesting aspect in the current study is that the tachykinin system becomes involved in a process that occurs bilaterally in response to a unilateral experiment. There was thus an upregulation of NK-1R expression bilaterally, as well as an increase in SP levels bilaterally as seen via EIA analyses. This suggests that the system has crossover effects. Similar crossover effects were reported in a recent study where retinal laser burn-induced neuropathy lead to an increase in SP-inducible NK-1R first in the retina of the burned eye and then in the contralateral eye [32]. The authors of this study, who had previously noted that unilateral retinal laser burn leads to inflammation not only in this eye but also in the nonburned eye [33], suggested that SP had transmitted early inflammatory signals from the affected eye to the contralateral eye. In the same context, Chang and collaborators [34] demonstrated that unilateral burn injury in a limb can have a long-lasting allodynia that spreads to the contralateral limb. It was concluded that central neuropathic mechanisms, via the occurrence of the hyperexcitability of second-order neurons and microglial activation, were responsible for the cross-transfer effect [34].

It has previously been shown that unilateral experiments and unilateral inflammation in limbs can lead to contralateral effects and in which cases neurological mechanisms are considered to be very important [35–38]. Occurrence of contralateral responses following unilateral stimuli in the form of intradermal injections of capsaicin has also been shown for humans [39] and contralateral increases in sensory neural activity are suggested to be of importance for the symmetry concerning the inflammation in rheumatoid arthritis [40]. It is furthermore known that electrical stimulation can lead to orthodromic activation of afferent nerve fibers which can initiate the release of SP and changes in both the stimulated and the nonstimulated contralateral muscles [41]. Bilateral effects concerning SP have previously been seen after experiments using heat injury [42] and unilateral formaldehyde injections [43] and in response to craniofacial inflammation [44] as well. It should here be stressed that the NK-1R expressions seen in our model of myositis/muscle injury were not only related to nerve structures but also inflammatory cells, blood vessel walls, and morphologically abnormal muscle fibers. It is also noteworthy that SP in experimental studies has been found to have a systemically acting wound messenger effect in the early wound healing processes after an injury to the conjunctiva [45]. In future studies using the currently used model, the bases for the contralateral effects should be further evaluated. It should be recalled that affections occurred bilaterally for the tendons in studies focusing on the Achilles tendons using the unilateral setup utilized in the present study [20].

### 4.3. Presence of NK-1R in Axonal Profiles and Large Nerve Fascicles

It is well known that there is a widespread expression of NK-1R in the central nervous system. It is also shown that unmyelinated axons in nerve bundles in peripheral locations can display NK-1R [46], and that NK-1Rs are found on sensory nerves in various locations [47, 48]. However, nerve-related NK-1R immunoreactions were very infrequently seen in the controls and areas showing normal muscle morphology in experimental animals in this study. The only profiles that were encountered occurred as varicose profiles.

On the other hand, nerve-related NK-1R immunoreactions were frequently detected in myositis areas and areas that were very close to these areas. NK-1R immunoreactions were thus seen in the form of fine distinct point-like reactions that were coalesced in large nerve fascicles and in the form of strong immunoreactive axonal profiles. The double stainings we performed showed that these NK-1R immunoactions were often associated with markedly S100beta immunoreactive Schwann cells. This observation suggests that these NK-1R expressing profiles are enclosed by Schwann cells. In accordance with this interpretation, previous studies made at the ultrastructural levels in the rat dental mucosa have shown that NK-1Rs are present in vesicular structures and the axoplasm of axons that are enclosed by Schwann cells [49]. Our double stainings also showed that the large nerve fascicles/axonal profiles harbouring these NK-1R reactions were related to nuclei that showed another type of fluorescence reactions than nuclei located outside the nerve fascicles and than those usually seen for nerve fascicles of normal muscle regions. The former fluorescence reactions are related to a combination of the DAPI reaction and S-100beta immunoreaction. It, therefore, seems likely that these nerve fascicles/axons are in a phase of regeneration. It is thus known that the S-100beta protein in principle is detected in the cytoplasm and the membranes of the Schwann cells, and not the nuclei, as seen in immunohistochemical stainings, in normal situations [50]. Furthermore, it is known that Schwann cells can be activated when there is a nerve damage and that the S-100beta protein can be involved in axonal regeneration [51, 52] and that Schwann cells ensheath regenerating axons [53].

### 4.4. Presence of NK-1R in White Blood Cells

The NK-1R, as well as tachykinins like SP, has been detected in several types of white blood cells [54]. In the present study, NK-1R was detected in macrophages and eosinophils. NK-1R was often colocalized with SP in the cells. The SP/NK-1R system may thus have an important role for the accumulation of these cells in the muscle tissue in the myositis process. Concerning macrophages, these findings are in accordance with a report saying that NK-1Rs are involved in the accumulation of macrophages in the airways in the development of smoking-induced emphysema and in response to cigarette smoking exposure [55].

The NK-1R reactions in the cells in the inflammatory infiltrates showed frequently an intracellular location. This is likely to be due to the NK-1Rs being internalized after binding to released SP or that it represents newly synthesized receptors. It is well known that the NK-1R in neurons undergoes rapid internalization after binding to SP has occurred and that the receptor thereafter recycles to the plasma membrane [56]. The intracellular location of NK-1Rs has been reported to be transport vesicles and endosomes [57]. Also in nonneuronal cells, there is a process of endocytosis and recycling of the NK-1R [58]. It might be that the cells in the inflammatory infiltrates are under the continuous influence of SP in an autocrine/paracrine fashion and that there is a continuous NK-1R internalization but also a continuous synthesis. SP and the NK-1R are actually reported to be internalized in the same vesicles and then sorted into independent endosomes, leading to the eventual degradation of SP and recycling of the NK-1R [59].

### 4.5. Presence of NK-1R in Blood Vessel Walls

NK-1R immunoreactions and NK-1R mRNA reactions were seen in the endothelium of blood vessels in myositis areas. It is well known that SP induces an increased vascular permeability, vasodilatation, and hyperthermia via binding to NK-1Rs on the endothelial cells of the blood vessel walls [60]. The vasodilator effect by SP is thus considered to be dependent upon the endothelium and to be mediated by the NK-1R. We also sometimes noted reactions for NK-1R mRNA for the smooth muscle layer. In accordance with this finding, it has been found that smooth muscle cells of airways in rats show mRNA transcripts for the NK-1R [61].

### 4.6. NK-1R in Degenerating/Regenerating Muscle Fibers

NK-1R was not only detected in white blood cells that were dispersed in the tissue but also in white blood cells that heavily had infiltrated muscle fibers. Based on the complete or relative loss of desmin immunoreactivity in these muscle fibers, they can be considered to represent degenerating/necrotic muscle fibers. It is thus known that desmin is undetectable or much decreased in necrotic muscle fibers [62]. On the other hand, we noted that other muscle fibers in myositis areas and adjacent areas showed a very marked desmin immunoreaction. These fibers are interpreted to be in a regenerative stage. This is in accordance with the known fact that there is an overexpression of desmin during muscle regeneration processes [63]. Also these fibers exhibited NK-1R immunoreaction, the reactions in this case showing a punctuate pattern. This indicates that both degenerative and regenerative events occur in the myositis process and that the NK-1R is involved in both types of processes.

### 4.7. Functional Aspects: General Considerations

As discussed above, there were marked NK-1R reactions in myositis areas and areas adjacent to these areas, whilst there were almost no NK-1R reactions at all in areas with normal morphology. The reactions were seen for nerve structures, white blood cells, blood vessel walls, and abnormal muscle fibers. The observations of a very marked NK-1R expression in myositis specimens, in parallel with an increased content of SP in these specimens (as seen for specimens of the 6-week groups), are comparable to findings made in other situations for tissues exhibiting inflammation and tissue damage/reorganization. That includes colonic fibrosis [64], immunodeficiency virus lesions [65], airway inflammation [66], and sarcoidosis [67]. It has since long been considered that the occurrence of interactions between tachykinins and inflammatory cells is of great importance in pathological conditions and that the NK-1R has important roles in these conditions [68]. There is a reason to believe that tachykinin effects hereby are related to interactive effects with other signal substances.

The fact that SP and NK-1R sometimes were found to be colocalized suggests that autocrine/paracrine SP effects may occur in the tissue. This can be related to an auto-regulatory mechanism for SP in relation to the NK-1Rs. Autoreceptor functions for SP affecting NK-1Rs have since long been suggested to occur in other situations [46]. It has also previously been suggested that NK-1Rs found on sensory neurons are autoreceptors, being related to the modulation of peripheral pathophysiological effector functions [47]. Autocrine/paracrine effects by SP has on the whole been considered to take place in several conditions, including in leukemia [69] and various inflammatory situations [70].

One can ask wheter the marked NK-1R expression in the myositis areas is not only related to proinflammatory effects but also to healing effects. It should here be recalled that there occurs an overexpression of the NK-1R not only in response to inflammation and tissue damage, but also in situations with tissue repair and wound healing. That includes the situation during gastric wound healing in rodents [71]. An inflammatory component is actually described to be involved in healing effects for the skin. Topical treatment of SP to skin wounds in genetically diabetic mice thus leads to an increase in inflammatory density as a part of the healing process [72]. SP is considered to be involved not only in cutaneous healing, but also in corneal wound healing [3] and to be involved in processes of tendon repair [73–75]. Concerning tendinopathy, SP can accelerate the processes of hypercellularity and angiogenesis in tendon tissue in this condition [76]. It is furthermore considered that SP can have protective roles. It is, for example, reported that NK-1R-mediated functions have protective roles in acute hyperoxic lung injury [77].

## 5. Conclusions

The present study shows that the tachykinin system is markedly involved in the processes of muscle injury/myositis that occur in the overuse model here used. To what extent the effects of tachykinin are related to proinflammatory or tissue-healing effects remains to be answered. Our findings suggest that the NK-1R is involved in both degenerative and regenerative events. There is a reason to believe that tachykinins show interference effects with other signal substances in the processes that occur. The tachykinin system is upregulated on both sides, that is, in the myositis process that occurs in the experimental side as well as the one that occurs contralaterally. The upregulation is for both sides not only restricted to nerve structures but also to cells of the inflammatory infiltrates, the blood vessels, and morphologically changed muscle fibers.

It has not previously been demonstrated that tachykinins have the marked effects that are shown here for muscle tissue. The currently used model can be utilized in further studies evaluating the features of interactions between tachykinins and other signal substances in myositis and degenerative/regenerative muscle processes and to evaluate if interference with tachykinin effects can be of value for these processes. The possible effectiveness of NK-1R blocking has been evaluated for various inflammatory conditions [78, 79]. The results have been unclear. It remains for the future to evaluate if interference with effects at the NK-1R can have positive effects in situations with myositis and muscle injury.
